# Isolation of Primary Brain Cells: Challenges and Solutions

**DOI:** 10.26502/acbr.50170464

**Published:** 2025-07-14

**Authors:** Arnav Aggarwal, Yssel Mendoza-Mari, Anshu Aggarwal, Devendra K. Agrawal

**Affiliations:** 1 Loveless Academic Magnet Program, Montgomery, AL, USA; 2 Department of Translational Research, College of Osteopathic Medicine of the Pacific, Western University of Health Sciences, Pomona, CA, USA; 3 Department of Biology and Environmental Sciences, College of Science, Auburn University at Montgomery, Montgomery, AL, USA

**Keywords:** Astrocytes, Brain, Central nervous system, Microglial cells, Neurodegenerative diseases, Neurofilament proteins, Neurons, Primary cells, Stroke, Traumatic brain injury

## Abstract

The isolation of primary brain cells is essential for studying cellular behavior, signaling pathways, and disease mechanisms in the central nervous system. This paper explores the general and specific steps involved in extracting and culturing neurons, astrocytes, and microglia from brain tissue, highlighting how primary cells maintain their functionality and structural integrity without genetic modification like immortalized cell lines. Marker proteins such as MAP-2, GFAP, IBA-1, and TMEM119 help confirm cell identity and allow tracking of phenotypic changes, such as inflammation or maturation. We critically discussed some technological problems that researchers usually face during extraction and culturing procedures, emphasizing that each brain source and particular cell type require strict conditions to maximize cellular yield and viability. Environmental control of the cells in culture, such as pH, CO_2_, substrate coating and correct medium formulation, are critical for maintaining healthy and viable brain cell cultures. Limited lifespan and sensitivity of primary neurons restrict long-term experiments and increase the risk of experimental variability. Batch-to-batch variation in tissue sources leads to inconsistency in phenotype and function, especially with primary cell isolations. Ethical and practical limitations in sourcing human brain tissue reduce the generalization of findings and force reliance on clinically relevant experimental animal models that represent human conditions.

## 1. Introduction

Neurological disorders that affect the nervous system, including the brain, spinal cord, and nerves, are the leading cause of morbidity, disability, and mortality worldwide. In traumatic brain injury, concussion, and stroke, diffuse axonal injury, loss of neuronal and glial cells, brain edema, and ischemic brain damage after primary brain injury contribute to secondary brain injuries resulting in cognitive deficits, behavioral changes, and hemiparesis [1–5]. Increased oxidative stress, excitotoxicity, mitochondrial dysfunction, lipid peroxidation, neuroinflammation, axonal degeneration, inhibition of myelination and axonal growth, increased apoptosis of neurons and oligodendrocytes, and impairment of autophagy and lysosomal pathways contribute to secondary brain injuries [6–10]. Ischemia, infarction, and resulting inflammation in the brain greatly affect patient outcomes and can be modulated with prompt recognition and treatment. Several pre-clinical studies identified potential targets in the treatment of neurological disorders, and electromagnetic field stimulation (EMF) has been found to be effective in the healing of injured and ischemic areas in the brain [11–17]. However, underlying mechanisms of action of many therapeutic strategies, including EMF are unclear, and warrant further investigation on their effects on differentially expressed genes and proteins, and various intracellular organelles and their functions using the individually purified primary brain cells.

Therefore, the isolation of brain cells is very important for conducting precise and controlled experiments, as it allows researchers to focus on specific cell types and observe their responses without interference from surrounding tissue. This improves accuracy in studying cellular functions and gene expression. Results could be inconsistent or misleading without isolation due to mixed cell populations.

There are several immortalized cell lines commercially available that were derived from primary cells or tumors of different origins and species. These cell lines are less expensive to acquire, compared to primary isolations, and they are easy to culture and expand, allowing large-scale experiments with high reproducibility. Nevertheless, the genetic modification disrupts their normal physiological functioning, making them significantly different from primary cells, and inappropriate for several applications [18]. Due to their capacity to undergo multiple cell divisions, they may accumulate mutations over time [19]. On the other hand, numerous cell lines are derived from embryonic tissues, so they might not exhibit the adult phenotype and behavior; therefore, the interpretation of the results obtained after using them must be carried out with caution.

Primary cells allow researchers to conduct experiments quite like the *in vivo* environment. These cells typically retain the characteristics of the original tissue, making them useful for experimental studies under controlled conditions and for translating the results to pre-clinical and clinical scenarios. However, primary cells have a limited lifespan, undergoing senescence after a few divisions, and often require specific growth factors and cell culture conditions. The isolation process usually takes time and can be expensive, according to the selected protocol, and every step might represent a potential source of contamination. Additionally, although following the same procedure, each isolation may not render identical results to the previous one, so a phenotypic characterization of each batch is required to avoid or minimize the inconsistencies from one experiment to the other.

Hence, isolating primary cells from animal or human tissues is always preferable, but this will require several sample sizes based on the healthy *vs* disease conditions from the source of the original tissue. The proper sample size to draw a meaningful conclusion from the findings must be determined using a power analysis. While testing novel compounds, it is very important to consider the sex-based differences in pharmacological response, pharmacodynamics, and pharmacokinetics [20], as it has been demonstrated that women are 50–75% more likely to experience adverse reactions to prescription medication [21]. On the other hand, there is also an age-dependent activity; aged neurons have different characteristics and response capacity than embryonic or young cells [22]. Finally, another aspect to contemplate is the difference between human and murine cells. Therefore, it is highly recommended to carry out experiments on human isolates when ethically possible, or failing that, on cells isolated from animals closer to humans, such as pigs or monkeys.

In summary, age, gender, and species must be taken into consideration before designing experiments based on primary neuronal cells to minimize the gaps and successfully translate the *in vitro* results to pre-clinical and clinical scenarios.

## 2. Isolation of Primary Brain Cells

**Figure 1:**
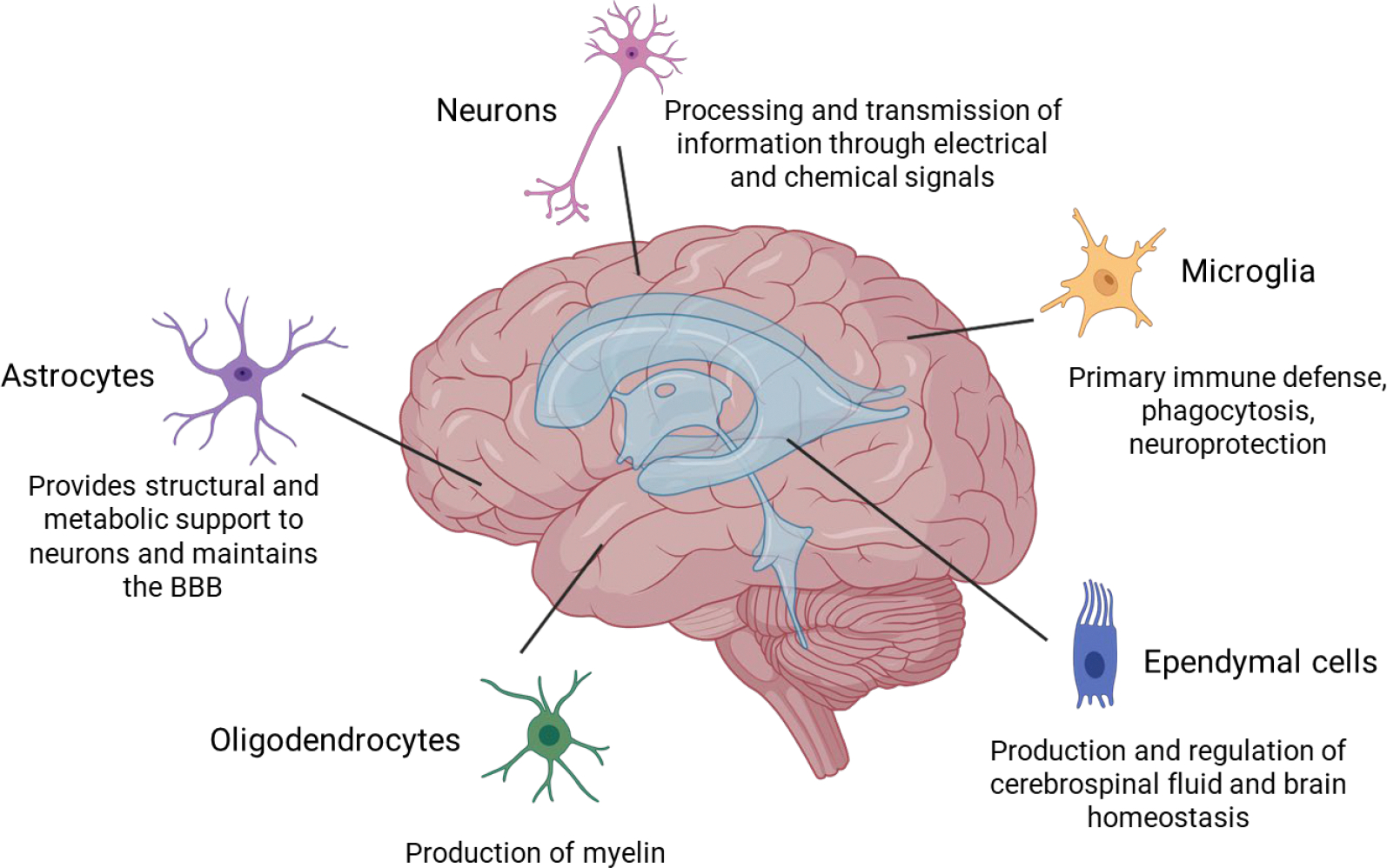
Major brain cell types: neurons, astrocytes, oligodendrocytes, microglia, and ependymal cells. Neurons transmit signals through chemical and electrical signals. Astrocytes maintain extracellular ion balance, regulate cerebral blood flow, and support the blood–brain barrier (BBB). Oligodendrocytes produce myelin in the central nervous system. Microglia provide immune defense and clean debris. Ependymal cells produce and regulate cerebrospinal fluid and brain homeostasis.

There are many different types of cells within the brain. Neurons and glial cells are among the most well-known and representative cells (Figure 1). Neurons are the main components of nervous tissue. They are responsible for processing and transmitting information through electrical and chemical signals from the presynaptic neuron to the target cell through the synaptic gap. Glial cells are non-neuronal cells that do not produce electrical impulses, but are crucial in maintaining homeostasis, forming myelin, and providing support and protection for neurons. They also play a role in neurotransmission and synaptic connections [23]. The neuroglia comprises microglia, astrocytes, oligodendrocytes, and ependymal cells. Microglia constitute approximately 5–10% of the total cells within the brain, and they serve as the primary immune defense in the central nervous system (CNS). They also participate in phagocytosis, removing dead neurons and cellular debris, and in neuroprotection [24]. Astrocytes are glial cells that possess numerous functions in the brain, such as maintenance of extracellular ion balance, regulation of cerebral blood flow, biochemical control of endothelial cells that form the blood–brain barrier (BBB), provision of structural and metabolic support to neurons and repair and scarring process of the brain and spinal cord following infection and traumatic injuries [25]. Oligodendrocytes are found in the white matter regions of the CNS, and their main function is to produce myelin to insulate axons [26]. Ependymal cells are located along the walls of the brain’s ventricles and play a crucial role in the production and regulation of cerebrospinal fluid (CSF) and brain homeostasis [27]. The information in this article is focused on current protocols for isolating microglial cells, astrocytes, and neurons.

**Figure 2:**
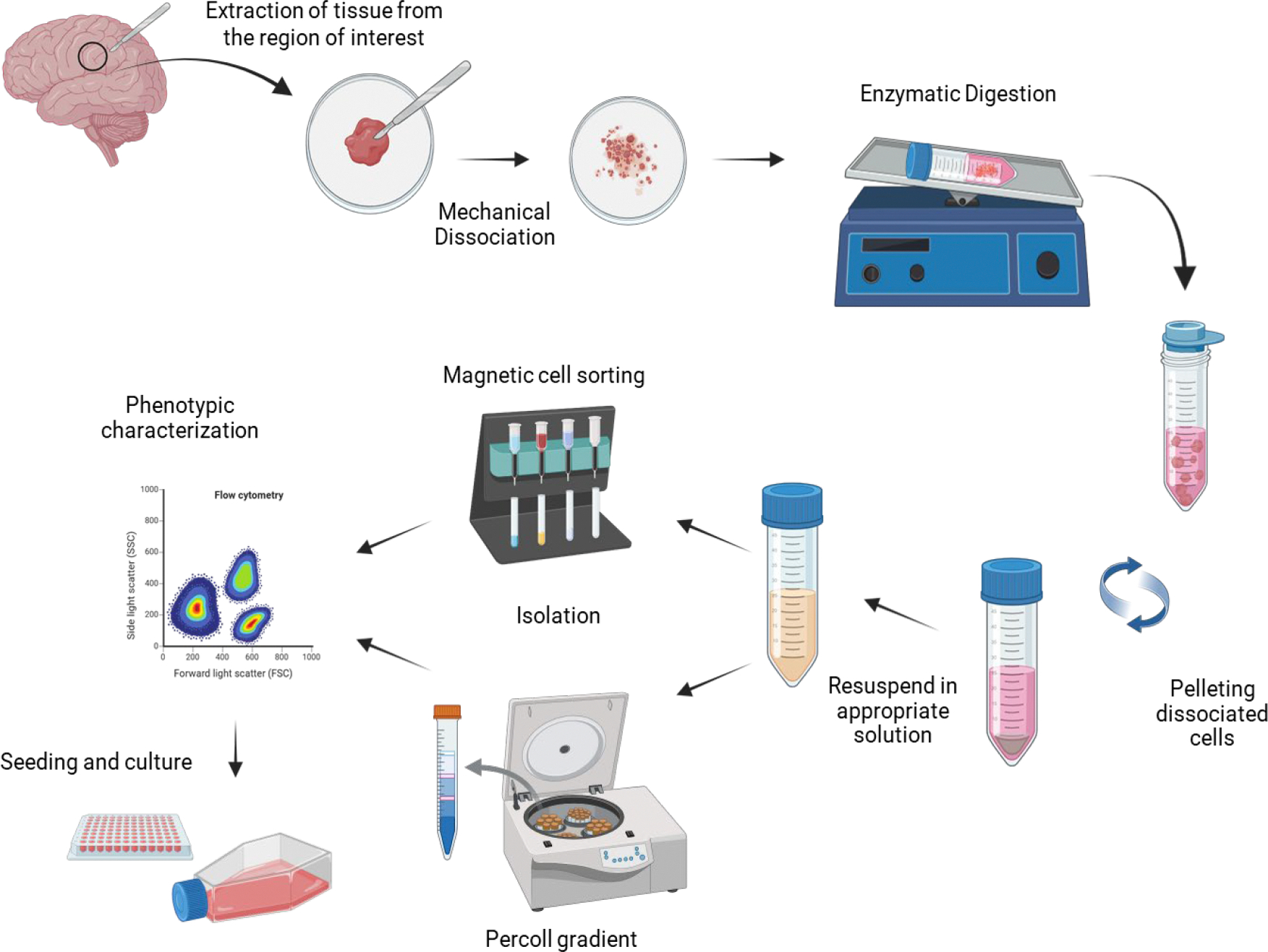
Schematic representation of general steps for isolating microglia, astrocytes, and neurons from the brain. A fragment of tissue from the region of interest is collected and aseptically transferred to a sterile area, where it is mechanically dissociated. The minced tissue is enzymatically digested to enhance dissociation. After incubation at 37°C, the tissue is filtered through a 70 μm cell strainer to remove clumps, then centrifuged to discard cellular debris present in the supernatant. The cell pellet is resuspended in an appropriate solution according to the selected isolation protocol. Magnetic cell sorting or Percoll gradient are among the most common procedures used to purify the different cell types. Fractions are characterized by flow cytometry or immunocytochemistry and seeded in the proper culture medium. Created using BioRender.com.

Isolation of these primary brain cells involves several common steps, including dissection, mechanical disruption, and enzymatic digestion, to obtain a single-cell suspension. Brain tissue is carefully dissected from the skull in each region, depending on the experimental goals (e.g., prefrontal cortex, thalamus, hippocampus). Next, the meninges, which are protective layers around the brain, are removed to expose the desired area to prepare for extraction. In the enzymatic digestion phase, enzymes such as trypsin facilitate cell separation by digesting intercellular proteins. After dissociation, the protease is inactivated, and the tissue homogenate is filtered through a cell strainer to remove cell clumps. Then, the cell suspension is centrifuged to remove cellular debris present in the supernatant, and finally, the cell pellet is resuspended in an appropriate solution, according to the isolation protocol selected. Immunocapture using magnetic beads or Percoll gradient are among the most employed methodologies to separate each cell type (neurons, oligodendrocytes, astrocytes, and microglia) (Figure 2).

## 3. Isolation of Multiple Cell Types from the Same Brain Tissue

### 3.1 Immunocapture using magnetic beads

This protocol is based on the use of magnetic beads conjugated to antibodies that recognize cellular surface markers that are specific for each type of cell. The interaction of these antibodies with their respective target molecules allows for the physical separation of the bound cells in the presence of a magnetic field. The general methodology includes the incubation of the cell suspension with the selected magnetic beads/antibodies, exposure to a magnetic field to retain positive cells inside the separation column and allow negative cells to pass through. Positive cells are washed several times to eliminate non-specific bound cells and are finally flushed out by removing the separating column from the magnetic field and pushing the plunger into the column. Negative cells collected from the pass and washing steps can be exposed to additional separation procedures using different cellular markers according to the cell type of interest.

For the isolation of microglia, astrocytes, and neurons in that order, there is a well-established tandem protocol based on the use of CD11b, ACSA-2 (astrocyte cell surface antigen-2), and a non-neuronal cell biotin-antibody cocktail magnetic bead, respectively [28]. The first step includes the collection of microglial CD11b+ cells. This marker, also known as integrin alpha M or ITGAM, is a surface protein commonly used to recognize microglia and other myeloid lineage cells, such as monocytes and macrophages. From the negative fraction, astrocytes are subsequently purified using microbeads conjugated to ACSA-2 antibody, which recognize this specific cell surface marker. Finally, CD11b/ACSA-2 negative cell suspension is incubated with a non-neuronal cell biotin-antibody cocktail magnetic beads that allow the depletion of remaining non-neuronal cells from the cellular mix and the purification of neurons by negative selection. This protocol is described for 9-day-old mice and, in general, the recovery and purity of different populations are high. According to the authors, some aspects that must be taken into consideration to increase the yield are the age of the mice and whether they are genetically modified or not. Once the cells are isolated, the other putatively limiting issue is the time in culture, as some cells start to change their morphology shortly after purification [28], highlighting the need to perform further experiments as fast as possible.

### 3.2 Percoll gradient

The Percoll gradient method is a density-based centrifugation technique used to isolate specific cell types from a mixed population, such as dissociated brain tissue. Agalave et al. [29] designed a protocol that allows the isolation of primary microglia and astrocytes from rodents CNS [29]. This methodology is intended to circumvent the use of expensive fluorescent antibodies or immunomagnetic beads and to avoid the enzymatic digestion, which might affect cell viability due to mild toxicity or damage cell surface epitopes [30]. The Percoll gradient is prepared using 70%, 50% and 35% stock isotonic Percoll solutions. Briefly, brain tissue is mechanically disrupted and filtered through a 70 μm nylon cell strainer. Pelleted cells are resuspended in 70% stock isotonic Percoll solutions, and afterwards, lower percentage stock isotonic Percoll solutions are carefully allowed to layer over to form the gradient. After centrifugation without brake, astrocytes can be recovered in the interface between 35% and 50% stock isotonic Percoll solutions, and microglia form a ring in the interface between 50% and 70% stock isotonic Percoll solutions. Once the cells are washed, they are immediately characterized and seeded for further experiments. One of the main difficulties encountered when using this method is preparing the Percoll gradient. Stock isotonic Percoll solutions of different densities are very difficult to differentiate with the naked eye, so great care must be taken when placing each one on top of the other to avoid mixing them. Likewise, the conical tubes containing the gradient must be handled carefully before and after centrifugation to prevent altering the distribution inside.

## 4. Other Protocols for the Isolation of Individual Cell Types

### 4.1 Primary microglia

Several protocols are described for the isolation of microglial cells from murine origin. Some of them use digestion cocktails containing collagenase and dispase, and discontinuous Percoll gradient separation [31]. Another procedure combines papain digestion, discontinuous Percoll gradient, and a subsequent culture selection process to collect microglial cells from a mixed glia population [32]. Primary microglia from post-natal 3-day-old rats can be isolated by their capacity to adhere to Aclar plastic film, a flexible thermoplastic fluoropolymer film with high optical clarity [33]. According to the authors, this methodology can be extrapolated to rat embryos and adult brains. Vijaya et al. [34] compared three different methodologies to isolate microglial cells from aging mice in terms of cell yield, purity, and functional properties of the purified cells [34]. According to their results, the optimal protocol comprises enzymatic digestion and the use of a debris removal solution. Besides, they seed the cells in appropriate culture medium for three hours and then remove the unattached cells, supplementing the isolated cells with macrophage colony-stimulating factor and granulocyte-macrophage colony-stimulating factor. Recently described methods utilize commercially available solutions to achieve mechanical and enzymatic dissociation of the brain tissue, as well as to obtain pure populations of primary microglia [35].

### 4.2 Primary oligodendrocytes

As expressed before, most of the protocols described to date are developed to purify primary cells from murine brains. The isolation methodologies employing robust laboratory species (swine, monkey) and human brain face important ethical and technical limitations. To warrant high rates of yield and viability, it is crucial to minimize the time lapse from euthanasia/death to tissue collection. The method described by De Groot et al. [36] is suitable for the isolation of microglia and oligodendrocytes from postmortem human brain tissue [36]. Microglial cells are isolated by a protocol like those described before. Meanwhile, oligodendrocytes derived from the adult brain are collected from the culture supernatant 24 hours after the initial plating, as they do not adhere to the surface of the cell flask. After, myelin debris is removed by a density gradient in a Lymphoprep solution, and the resultant interphase is washed and seeded in uncoated tissue flasks.

Oligodendrocytes can be isolated from mechanically and enzymatically dissociated tissues using magnetic activated cell sorting (MACS). Depending on the type of cell needed, beads can be conjugated to different antibodies: platelet-derived growth factor receptor (PDGFR) alpha or A2B5 (a monoclonal antibody binds to a specific ganglioside epitope present on the surface of oligodendrocyte precursor cells) are employed to isolate oligodendrocyte precursor cells; antibodies recognizing O4 (a surface lipid sulfatide), which is expressed by pre-myelinating oligodendrocytes, are used for this specific stage of oligodendrocyte development, while antibodies recognizing myelin oligodendrocyte glycoprotein or myelin binding protein are used to purify mature oligodendrocytes.

## 5. Primary Neurons

Unlike immortalized cell lines, primary neurons maintain the complex morphology and characteristics of neurons found *in vivo*. Because of this, they are widely used in neuroscience research for studying development, neurodegenerative diseases, synaptic plasticity, and responses to drugs or toxins [37]. One of the key aspects to keep in mind while isolating neurons is that, once in culture, they should maintain their functional properties to enable electrophysiology, calcium imaging, or synaptic studies. To achieve this, purification protocols should include the use of gentle enzymes like papain to minimize damage, the addition of DNase I to reduce clumping from released DNA, and the incubation time and temperature should be carefully optimized to avoid over-digestion, while mechanical disaggregation should be moderate to prevent shearing of neurites. The use of Percoll density gradient centrifugation before applying more specific methods reduces microglial and astrocytic contamination to enrich viable neurons.

According to the studies that will be performed, it will be preferred to work with embryonic or neonatal tissues due to their higher neuronal plasticity and survival. While working with adult tissues, researchers should take into consideration that they require more rigorous enzymatic digestion and may yield fewer viable neurons. Once a neuronal-enriched semi-purified cell suspension is obtained, a method based on MACS can be performed. Different intracellular markers are used according to the desired specific neuronal subtype: neuronal nuclear protein (NeuN) for general neurons, class III β-tubulin for early neurons, tyrosine hydroxylase for dopaminergic neurons, glutamate decarboxylase 67 for GABAergic neurons, choline acetyltransferase for cholinergic neurons, vesicular glutamate transporter 1 for glutamatergic neurons, among others. Also, surface markers are useful for neuronal isolation: Neural cell adhesion molecule or CD56 as a general neuronal marker, CD24 for developing neurons, CD133 or prominin-1 for neural progenitors, or the neurotrophic tyrosine kinase receptors TrkA/B/C for sensory neuron subtypes. After purification, neurons should be seeded on poly-D-lysine or laminin-coated surfaces to promote adhesion and maintained in proper culture medium complemented with B27 or N2 supplements to warrant high survival levels.

A specific protocol to isolate functional adult neurons from neurosurgical human brain sections was described by Park et al. [38]. Tissue fragments were transported to the laboratory in a specific ice-cold medium to ensure preservation. Enzymatic digestion was carried out in the presence of papain/DNAse I, and undigested tissue fragments were separated by a 100 μm cell strainer filtration step. Cells were cultured in Dulbecco’s Modified Eagle medium/F12 supplemented with B27, the Rho-associated coiled-coil containing protein kinase (ROCK) inhibitor Y-27632 dihydrochloride, and a cocktail of neurotrophic factors. With this methodology, it was possible to maintain the cells for at least 28 days *in vitro*, during which neurons recovered their neurite processes and electrophysiological properties, allowing the authors to perform studies about synaptic connections [38].

## 6. Analysis of the Purity, Phenotype and Viability

After the isolation of the different cellular types from the brain, it is necessary to assess the purity of cultures obtained, especially if we are working with mixed isolations. These studies are essential to ensure that the isolations contain the intended cell type and that contamination from others unwanted cells is minimal. Cellular characterization can be accomplished through different methodologies, most of them based on specific cell markers. For example, immunocytochemistry or immunofluorescence is the gold standard method for cell-type identification. It allows the analysis of purity by counting the number of positive cells to a specific marker under a fluorescence microscope. On the other hand, flow cytometry allows a reliable quantitative analysis of cell populations using fluorescently labeled antibodies. Among the advantages of this technique are that it can assess multiple markers simultaneously and that it is useful for both surface and intracellular markers, after permeabilization of the cells. Isolations can also be characterized employing more traditional techniques like quantitative real time PCR and Western blot. Both provide a molecular-level purity profile and allow the extrapolation from the transcriptional to the translational level. Researchers can also perform the morphological assessment of the isolations, although this is only a qualitative, not quantitative, analysis.

**Figure 3:**
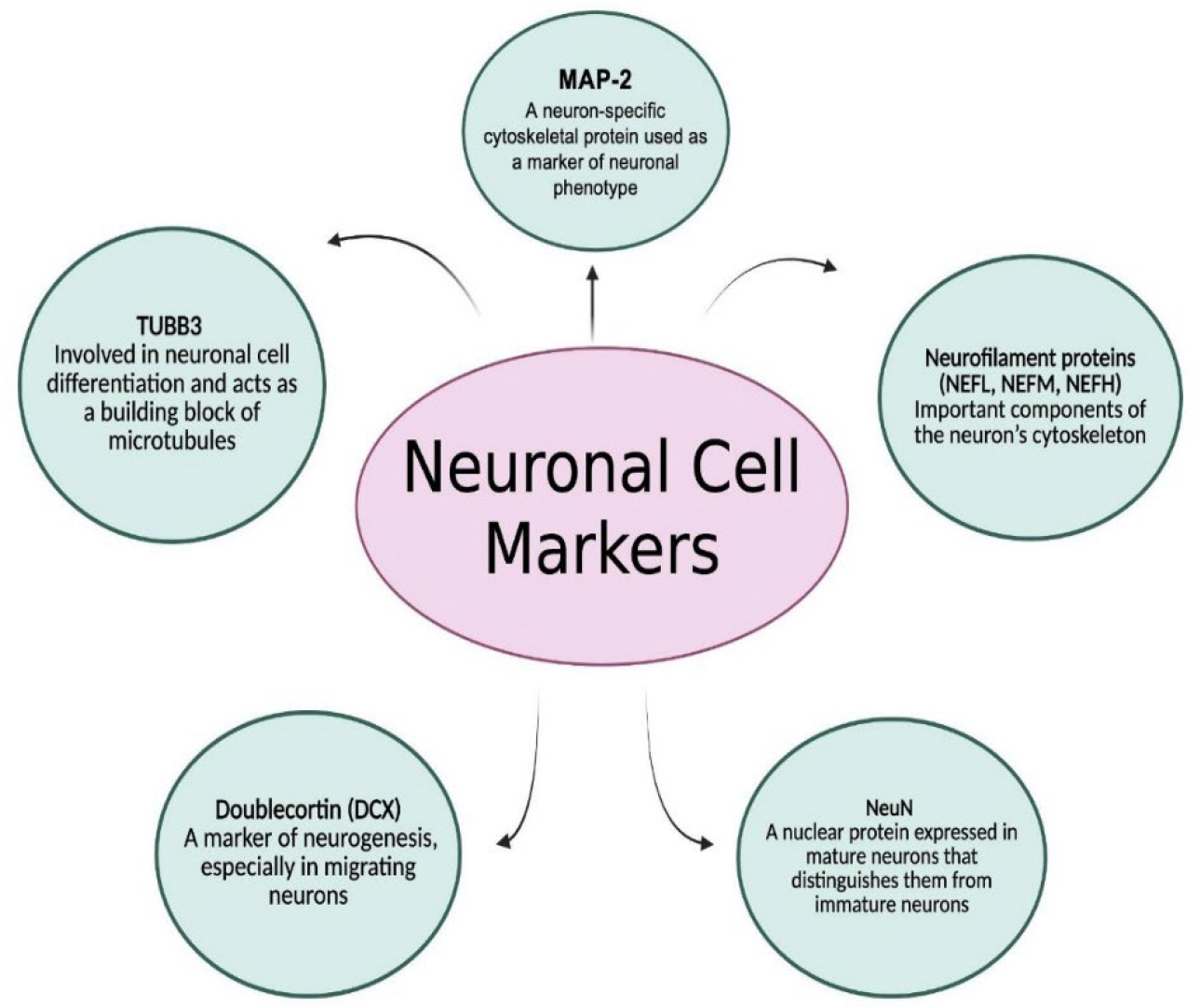
Common markers used to identify and study neurons. These include MAP-2 (microtubule-associated protein 2), a cytoskeletal protein indicating neuronal phenotype; TUBB3 (Tubulin beta-3), essential for differentiation and microtubule formation; DCX (doublecortin), a marker of neurogenesis in migrating neurons; NeuN (Neuronal nuclei), specific to mature neurons; and neurofilament proteins (NEFL, NEFM, NEFH), which are vital components of the neuronal cytoskeleton. These markers help distinguish neuronal subtypes and developmental stages in research. Created using BioRender.com.

As expressed before, neuronal phenotypes are diverse, and various subtypes are distinguished by their connectivity patterns, neurotransmitter release, and overall functional roles. General markers to identify neurons comprise microtubule-associated protein 2 (MAP-2), tubulin beta III (TUBB3), doublecortin (DCX), neuronal nuclei (NeuN), and neurofilament proteins (NEFL, NEFM, NEFH) (Figure 3). MAP-2 is a neuron-specific cytoskeletal protein used as a marker of neuronal phenotype. TUBB3 is involved in neuronal cell differentiation and acts as a building block of microtubules. DCX is a marker of neurogenesis, the process of generating new neurons from neural stem cells, particularly in migrating neurons. NeuN is a nuclear protein expressed in mature neurons and helps distinguish them from immature neurons, while neurofilament proteins are essential components of the neuron’s cytoskeleton (Figure 3).

Glial cells are highly variable and adapt to different brain regions and developmental stages. For example, astrocytes may adopt different phenotypes, influencing their ability to help mediate neuroinflammation or support neuronal function. Glial cells may also exhibit subtypes within their broader categories. For example, astrocytes may be classified as active (reactive) or resting (quiescent) depending on their activation state. The developmental origin of a cell (ontogeny), injury or inflammation, which leads to reactive astrocytes or microglia, the connections a cell makes with other neurons, and external stimuli and signals are all factors that may influence the cell’s phenotype.

**Figure 4:**
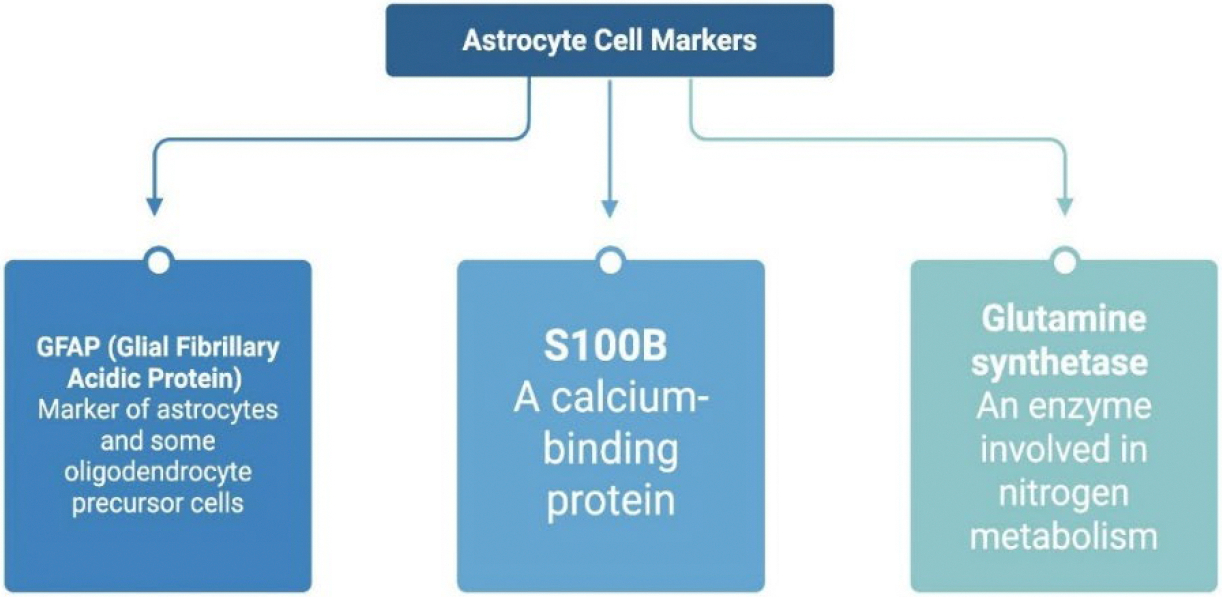
Key markers used to identify astrocytes, a primary type of glial cell. Glial Fibrillary Acidic Protein (GFAP) targets astrocytes and some oligodendrocyte precursor cells. S100B is a calcium-binding protein commonly found in astrocytes. Glutamine synthetase is an enzyme in nitrogen metabolism and indicates astrocytic metabolic activity. These markers help define astrocyte identity, function, and activation states in research. Created using BioRender.com.

Some astrocyte cell surface markers are glial fibrillary acidic protein (GFAP), S100B, and glutamine synthetase. GFAP is a marker of both astrocytes and some oligodendrocyte precursor cells (39). S100B is a calcium-binding protein, and glutamine synthetase is an enzyme with a key role in nitrogen metabolism (Figure 4).

**Figure 5:**
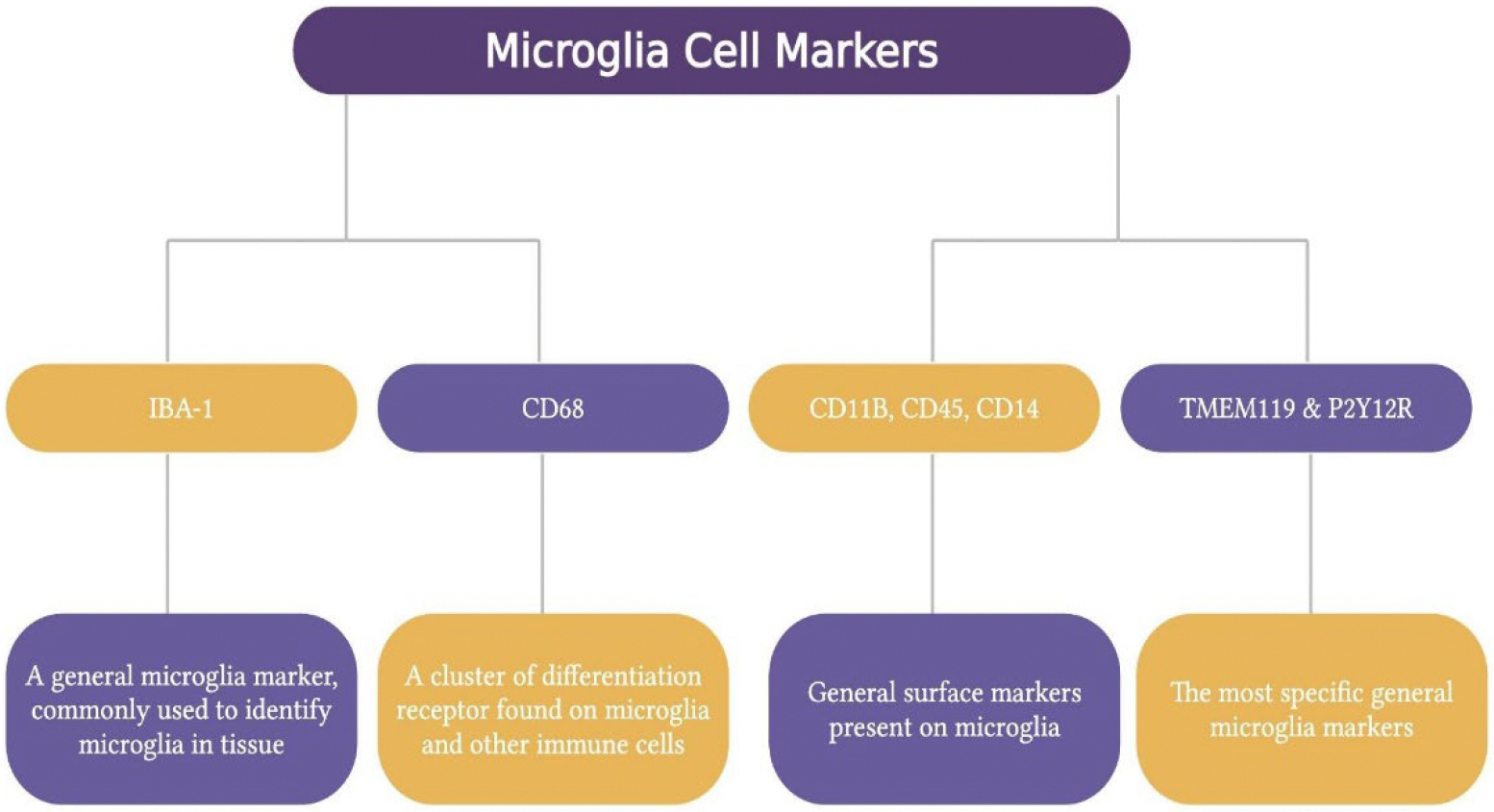
Markers used to identify microglia. IBA-1 is a widely used marker for detecting microglia in tissue. CD68, CD11B, CD45, and CD14 are general surface markers on other immune cells. TMEM119 and P2Y12R are the most specific general markers for microglia. These markers help distinguish microglial cells and are essential for studying their activation states (M1 pro-inflammatory and M2 anti-inflammatory). Created using BioRender.com.

Microglia markers are ionized calcium-binding adapter molecule 1 (IBA-1), cluster of differentiation (CD) 68, CD11b, CD45, CD14, CX3C motif chemokine receptor 1 (CX3CR1), and transmembrane protein 119 (TMEM119) and P2Y purinoceptor 12 (P2Y12R) [1]. IBA-1 is a general microglia marker most often used to identify microglia within the tissue. CD68 is a differentiation receptor cluster present on microglia and other immune cells, highly expressed during inflammatory processes. CD11b, CD45, and CD14 are other general surface markers, while TMEM119 and P2Y12R are considered the most specific general microglia markers [40]. Microglia can exist in different activation states, such as M1 (pro-inflammatory) and M2 (anti-inflammatory) phenotypes. These can be identified by their expression of specific surface receptors and release of soluble factors. For example, M1 phenotype is characterized by the expression of CD16, CD32, CD40 and CD86 and the secretion of pro-inflammatory cytokines such as interleukin (IL) 6, IL-17, IL-23 and tumor necrosis factor α. On the other hand, M2 phenotype can be distinguished by the expression of CD163 and CD206 and the secretion of IL-4, IL-10, transforming growth factor beta, insulin-like growth factor 1 and several neurotrophic factors [41] (Figure 5).

## 7. Problems and Solutions During Brain Cell Isolation and Culturing

The first challenge faced during the isolation of neuronal cells from human or animal brains is to minimize the contamination during the extraction of brain fragments. Most of the protocols are described for small animals like mice or rats. Once the animals are euthanized, the head can be removed and placed inside a laminar flow where it is handled with sterile instruments and can be quickly placed in solutions or culture medium supplemented with antibiotics. However, this procedure is impossible when working with more robust species such as swine, or when the source comes from surgical procedures or autopsies performed on humans. Simply accessing the brain, requiring the removal of cranial bone using a saw, requires considerable manipulation time to reach the desired anatomical area and represents a significant source of infection. To minimize the proliferation of bacteria and other sources of contamination, the time from extraction to the beginning of the isolation procedure should be minimized as much as possible, less than one hour is desirable. Besides, samples should be transported in sealed plastic containers, preferably in ice. Park et al. [42] suggest the use of nutrient media Hibernate A supplemented with B27 [42].

Brain cell culture, although very valuable, has problems. Some include maintaining the correct environment, preventing contamination, adhering to the specific needs of different brain cells, and ensuring proper adhesion. There is a problem with improper pH levels or CO_2_ levels, as they can negatively impact cell viability and growth; however, one can avoid that by checking the incubator’s temperature, the CO_2_ levels (typically at 5%), and ensuring pH levels are controlled. Contamination can occur from bacteria, fungi, yeast, and mycoplasma, leading to inaccurate results. This can be avoided through using sterile techniques such as autoclaving equipment and media, testing cultures for contamination, and using a clean and controlled environment. Sometimes, cells may not adhere to the culture vessel surface, which leads to poor growth. The way to mitigate this problem is to coat the culture vessel with a matrix like collagen, poly-lysine, or fibronectin, depending on the cell type [43].

Other sources of contamination from brain samples are erythrocytes and pericytes. During the first steps of the procedure is highly recommended to remove meninges and visible blood vessels from the brain sample. For erythrocyte elimination, tissue homogenates can be resuspended in erythrocyte lysis buffer allowing the process to take place in ice for 15 minutes. After this time, cells are centrifuged and resuspended to continue the isolation process. Pericytes are embedded in the membranes of blood micro vessels in the brain and play key roles like regulation of the BBB permeability, neuroinflammation, angiogenesis, and clearance of toxic metabolites, among others. Although required for specific studies, sometimes they are “contaminating” other types of cells, reaching up to 96% of mixed cultures, as they are mitotically active [44]. Pericytes can be eliminated during neuronal cells isolation by MACS, targeting their classical surface markers PDGFR beta, alanine aminopeptidase, CD13 or neuroglial antigen 2.

**Figure 6:**
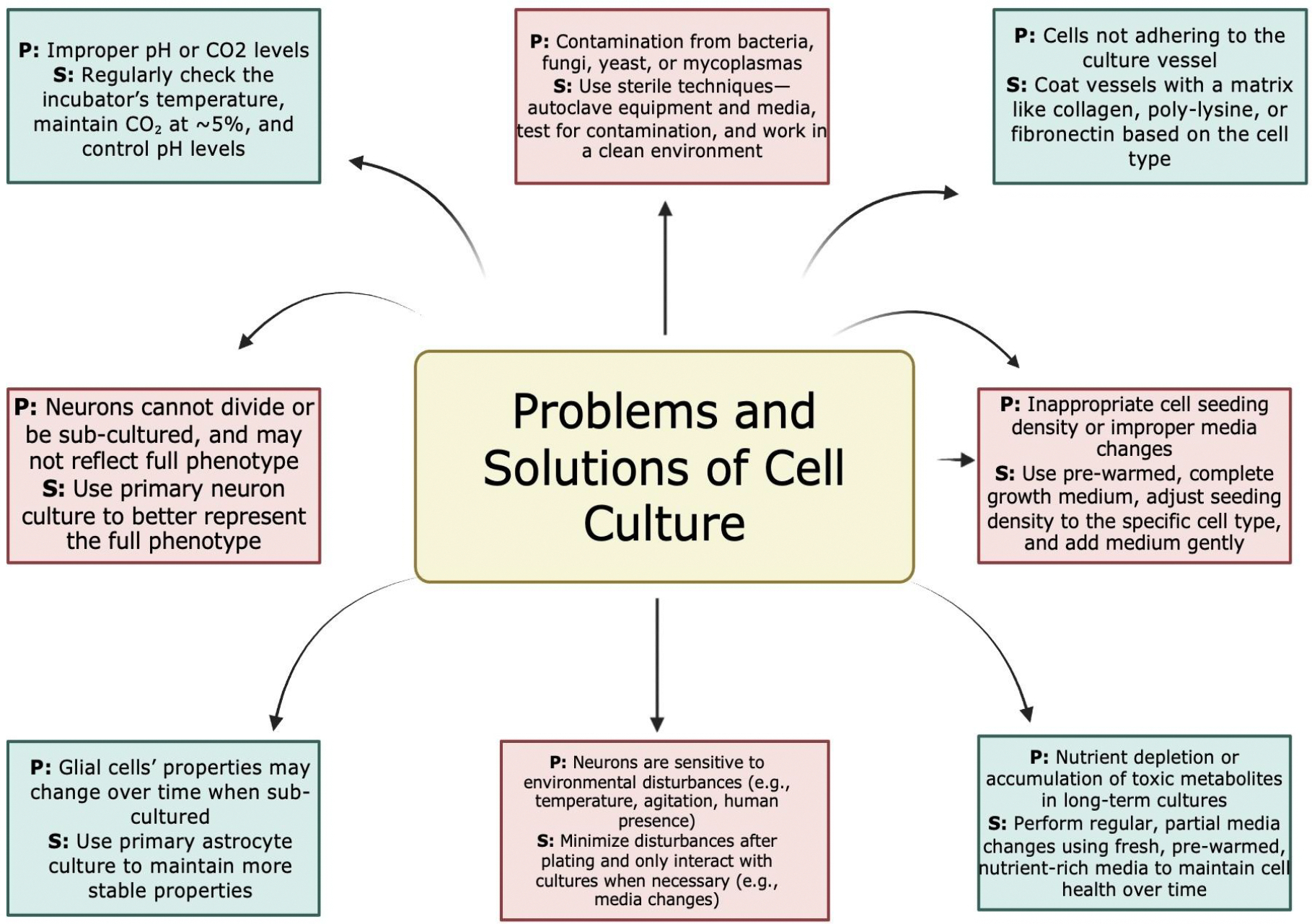
General problems (P) that can arise during the isolation of neuronal cells from human and animal sources. Solutions (S) involve environmental control, sterile techniques, appropriate substrate coating, minimizing disturbances, and regular media maintenance.

Low cell yields can be the result of an incomplete tissue dissociation or an inefficient enzymatic activity. To increase recovery from the tissue fragment, the researchers should select the correct enzyme according to the biological source and optimize the concentration to be used and the time of incubation, trying not to exceed the extension of the digestion process to maximize the cellular viability. In this case, using of gentle pipetting and adding antioxidants and ROCK inhibitor to the culture medium also contribute to obtain high viability and functionality of the cells, especially of neurons which are highly sensitive. Specifically, the ROCK inhibitor Y-27632 is crucial to stabilize the cytoskeleton and promote neurite outgrowth. Figure 6 depicts general problems and solutions to take into consideration while isolating neuronal cells.

## 8. Conclusion

The isolation and culture of primary brain cells, particularly neurons, astrocytes, and microglia, are important for studying the complex cellular mechanisms of brain function and disease. These techniques allow researchers to examine specific cell types in controlled environments to help understand their neurodevelopment, neurodegeneration, and neuroinflammation functions. While primary cells closely mimic *in vivo* behavior and retain native phenotypes, they are also sensitive and prone to variability depending on the source tissue. Proper handling during isolation, including dissection, enzymatic digestion, and careful separation, is important to avoid contamination and maximize cell viability and purity. In addition to develop a correct methodology for isolation, adequate culture conditions are crucial to extend lifespan and functionality of the cells. Extra challenges may be faced during isolation of brain cells from human and robust laboratory species like swine. While immortalized cell lines may be more convenient and consistent, primary brain cells represent a better and more accurate option for neuroscience research. However, findings must be validated through *in vivo* studies and supported by appropriate sample sizes and biological variables, such as sex, age and health status.

## 9. Key Points, Outstanding Questions, and Challenges

- Primary brain cell isolation allows researchers to study neurons, astrocytes, and microglia in a controlled setting while preserving their *in vivo* characteristics.
- Immunomagnetic separation using specific markers allows for the purification of different brain cell types.
- Marker proteins such as MAP-2, GFAP, IBA-1, and TMEM119 help confirm cell identity and allow tracking of phenotypic changes, such as inflammation or maturation.
- Environmental control (pH, CO_2_, substrate coating) and correct medium formulation are critical for maintaining healthy and viable brain cell cultures.
- What sex-based differences exist in primary brain cell responses, and how should these be accounted for in *in vitro* experiments?
- To what extent do primary brain cultures reflect *in vivo* behavior, and how can researchers close the gap between them when modeling diseases?
- How can co-culturing systems simulate natural brain environments without sacrificing cell viability or purity?
- Limited lifespan and sensitivity of primary neurons restrict long-term experiments and increase the risk of experimental variability.
- Batch-to-batch variation in tissue sources leads to inconsistency in phenotype and function, especially with primary cell isolations.
- Maintaining purity without compromising cell viability is difficult, especially during mechanical disruption and enzymatic digestion.
- Ethical and practical limitations in sourcing human brain tissue reduce the generalization of findings and force reliance on clinically relevant experimental animal models that closely mimic clinical conditions.
